# Correction: Agache et al. Nail Ultrasound in Psoriasis and Psoriatic Arthritis—A Narrative Review. *Diagnostics* 2023, *13*, 2236

**DOI:** 10.3390/diagnostics14020194

**Published:** 2024-01-16

**Authors:** Mihaela Agache, Claudiu C. Popescu, Luminita Enache, Bianca M. Dumitrescu, Catalin Codreanu

**Affiliations:** 1 Department of Internal Medicine and Rheumatology, “Carol Davila” University of Medicine and Pharmacy, 050474 Bucharest, Romanialuminita.enache@reumatologiedrstoia.ro (L.E.);; 2 Clinical Center of Rheumatic Diseases, 030167 Bucharest, Romania

In the original publication [1], the following acknowledgment statement was not included: “Publication of this paper was supported by the University of Medicine and Pharmacy Carol Davila, through the institutional program Publish not Perish”. The authors state that the scientific conclusions are unaffected. This correction was approved by the academic editor. The original publication has also been updated.
